# Extracellular Vesicle- and Extracellular Vesicle Mimetics-Based Drug Delivery Systems: New Perspectives, Challenges, and Clinical Developments

**DOI:** 10.3390/pharmaceutics12050442

**Published:** 2020-05-11

**Authors:** Prakash Gangadaran, Byeong-Cheol Ahn

**Affiliations:** 1 Department of Nuclear Medicine, School of Medicine, Kyungpook National University, Kyungpook National University Hospital, Daegu 41944, Korea; prakashg@knu.ac.kr; 2 BK21 Plus KNU Biomedical Convergence Program, Department of Biomedical Science, School of Medicine, Kyungpook National University, Daegu 41944, Korea

**Keywords:** extracellular vesicles, extracellular vesicle mimetics, pharmaceuticals, drug loading, delivery

## Abstract

Extracellular vesicles (EVs) are small membrane-based nanovesicles naturally released from cells. Extracellular vesicles mimetics (EVMs) are artificial vesicles engineered from cells or in combination with lipid materials, and they mimic certain characteristics of EVs. As such, EVs facilitate intracellular communication by carrying and delivering biological materials, such as proteins, lipids, and nucleic acids, and they have been found to find organ tropism in preclinical studies. Because of their native structure and characteristics, they are considered promising drug carriers for future clinical use. This review outlines the origin and composition of natural EVs and EVM engineering and internalization. It then details different loading approaches, with examples of the drug delivery of therapeutic molecules. In addition, the advantages and disadvantages of loading drugs into EVs or EVMs as a drug delivery system are discussed. Finally, the advantages of EVMs over EVs and the future clinical translation of EVM-based drug delivery platforms are outlined.

## 1. Introduction

Extracellular vesicles (EVs) are nanosized membranous vesicles released from most kinds of cells [1,2,3]. On the basis of their biogenesis, release pathways, and size, EVs are classified into exosomes (also called small EVs), microvesicles (MVs), and apoptotic bodies [1,2]. The history of EVs dates back almost four decades. In 1983, Harding et al. and Pan et al. first described exosomes and exosome secretion, which expanded our knowledge of the endosome–lysosome pathway [4,5]. The authors founded the starting point for the EV concept, although EVs had been unintentionally reported earlier [6,7]. Harding et al. (1983) reported membrane-bound vesicles released by multivesicular endosome (MVE) exocytosis from rat reticulocytes [4]. A decade later, Raposo et al. (1996) and Zitvogel et al. (1998) demonstrated that B-lymphocytes and dendritic cells (DCs) release exosomes via a similar route [8,9]. A decade later, researchers suggested that different types of cells exhibit the same exosomes release route through MVE fusion with the cell surface (cell membrane) [10,11]. MV biogenesis involves the direct trafficking of biological cargo to the plasma membrane, from which it is released by the use of contractile machinery at the surface to allow for vesicle blebbing [12,13]. MVs are distinct from exosomes. Apoptotic bodies are larger vesicles secreted by a dying cell process called blebbing, which is mediated through actin myosin interaction [14,15,16].

EVs were originally believed to be a source of cellular dumping, or a way for cells to get rid of unneeded or unwanted material. However, EVs can carry various biological constituents, such as lipids, proteins, and nucleic acids, and they are considered cargo delivery systems for long-distance communication between cells [3]. Pathologic cells, including cancer cells, secrete specific EVs with different compositions; therefore, EV can be used as diagnostic tools for certain diseases and can also be monitoring tools for the progression of diseases [17,18,19]. EVs deliver functional biological materials to recipient cells [20,21,22], and, therefore, researchers are focusing on using them as drug delivery systems. The drug delivery system is gaining increasing attention because it shows potential for improving the target delivery of drugs that are challenging conventional free drug delivery methods as comes with side effects by damaging of healthy cells [23,24]. Several studies have reported advantages of using EVs as drug delivery systems in preclinical models, as they have a low toxicity, high targeting capacity, and slow clearance from blood circulation by escaping degradation [12,25]. Therefore, EVs are emerging as emerging potential candidates for drug delivery. Already, a few human clinical trials are underway using EVs from DCs for cancer therapy, and positive results have been reported with regard to the feasibility and safety of EVs [26,27].

Researchers are also developing extracellular vesicle mimetic (EVM) drug delivery systems as a possible alternative to EVs. EVMs are produced artificially by the extrusion of cells by micron-sized membranes for drug delivery [28,29,30,31]. EVMs can be generated on a large scale compared to naturally released EVs in a short period [29,32]. Drug loading becomes simple because drugs can be loaded into EVMs during extrusion, while EVs need additional procedures for drug loading [30].

This review comprehensively explains EVs, including their biogenesis, composition, and structure. It also elaborates on EV (exosomes and MVs) drug-loading technologies and applications in drug delivery with examples. In addition, it discusses EVM engineering and the disadvantages of using EVs and EVM, as well as possible solutions for future advancement and clinical developments.

## 2. Origin and Composition of Naturally Secreted EVs

**Figure 1:**
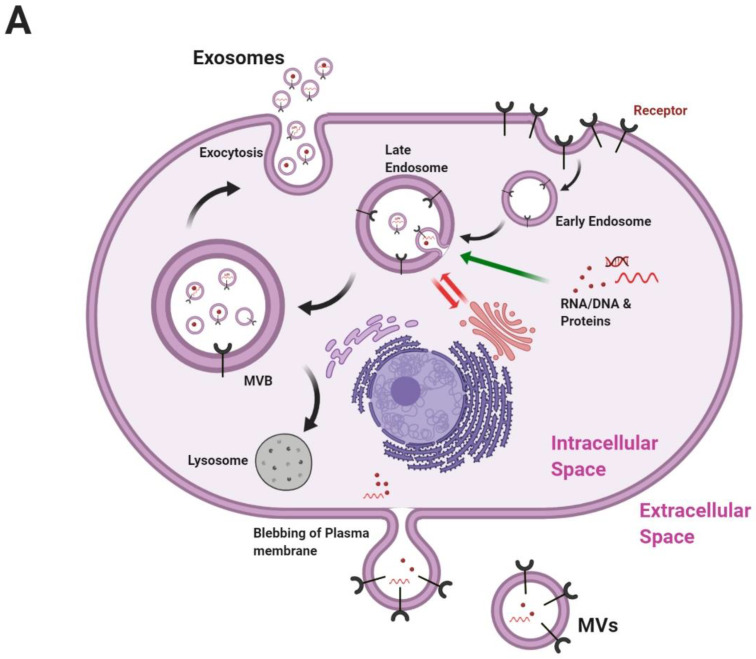

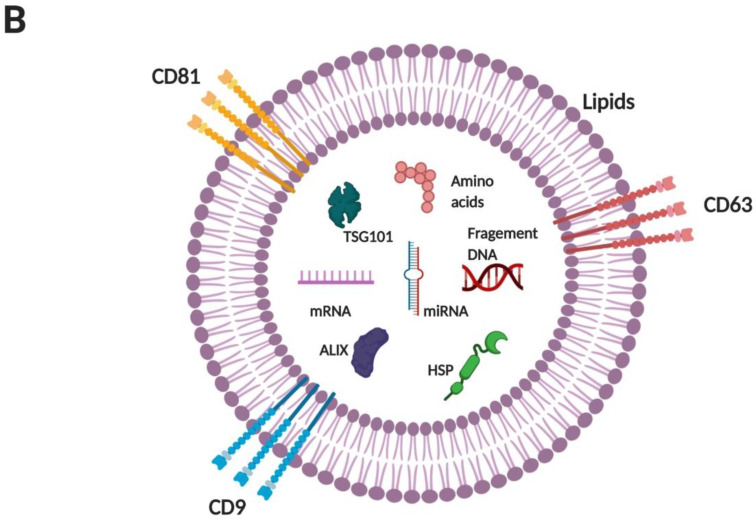
Biogenesis and composition of EVs. (**A**) Exosomes are generated by inward budding during endocytosis. Early and late sorting endosomes are assembled within MVBs, where specific exosomal cargos are sorted into exosomes. MVs are generated by plasma membrane budding. (**B**) A typical EV generally comprises proteins, lipids, RNAs, and genetic material. Proteins in EVs include transmembrane or lipid-bound extracellular proteins (CD63, CD9, CD81, etc.), cytosolic proteins (Alix, TSG101, etc.), intracellular proteins (HSPs). An EV also contains lipids (ceramide), RNAs (mRNA, miRNA, etc.), and DNAs (fragments). EV: extracellular vesicle; MVB: multivesicular body; MV: microvesicle; CD: cluster of differentiation; TSG101: tumor susceptible factor 101; HSP: heat shock protein; mRNA: messenger RNA; miRNA: microRNA. Figure created with BioRender.

EVs released from cells are heterogeneous and categorized on the basis of their origin and size (Figure 1) into exosomes, MVs, and apoptotic bodies. Exosomes are typically 30–150 nm in size because of a multivesicular body (MVB) [33]. Exosome formation starts from endocytosis, and exosomes are formed from the inward pushing of the plasma membrane. The endolysosomal system comprises a complicated and dynamic membranous network that transits from the early to late sorting of endosomes. Then, its forms MVBs and finally fuses with the plasma membrane for secretion [3]. MVB formation is of two types: endosomal sorting complexes required for transport (ESCRT)-dependent or ESCRT-independent.

The ESCRT (ESCRT-0, ESCRT-I, ESCRT-II, and ESCRT-III) system is the most widely reported mechanism for MVB formation. ESCRTs are accessory proteins that sequester ubiquitinated proteins into intraluminal vesicles (ILVs) [34]. Early acting ESCRT complexes (ESCRT-0, ESCRT-I, and ESCRT-II) harbor ubiquitin-binding domains, which play a key role in cargo selection [35]. Syntenin-1, Alix, and syndecan are proteins involved in ESCRT-III-dependent MVB formation, and heparinase promotes the cargo sorting of cluster of differentiation 63 (CD63)—but not CD9, CD81, or flotillin-1—into exosomes [36,37]. MV biogenesis is different from exosome biogenesis: molecular cargo is transported to the plasma membrane for budding and release, while exosomes are released from MVBs. MV biogenesis requires small guanosine triphosphatase (GTPase), such as adenosine diphosphate (ADP)-ribosylation factor 6, Ras-related protein Rab-22A, and acid sphingomyelinase (Figure 1) [38,39,40].

EVs generally comprise proteins, lipids, RNAs, and DNAs (Figure 1). Proteins constitute the major portion of EVs and are packed into EVs on the basis of the cells that secrete them and biogenesis. Exosomes tend to be more enriched in major histocompatibility complex class II (MHC class II) and tetraspanins (CD37, CD53, CD63, CD81, and CD82) [41]. The protein the composition of proteins changes on the basis of cell types [20,42,43,44,45,46]. MVs originate from the plasma membrane. They are mostly enriched in a different collection of proteins compared to exosomes, such as integrins, glycoprotein Ib (GPIb), and P-selectin [47]. MVs carry an abundant amount of proteins with posttranslational modifications (glycoproteins or phosphoproteins) compared to exosomes [48].

Lipids are derived from the plasma membrane by inward budding in for exosomes and by blebbing for MVs. EVs have abundant raft-associated lipids, such as ceramide, sphingolipids, phosphoglycerides, and cholesterol [49,50]. Phosphatidylserine is considered a lipidic signature of EVs. Lipids are important factors for drug delivery because they contribute to the excellent physicochemical stability of EVs, which enables EVs to directly merge with the plasma membrane of recipient cells.

Exosomes and MVs are produced and secreted during normal cellular activity; on the contrary, apoptotic bodies are released during apoptosis, which is one of major mechanisms of cellular death [14]. They are larger in size compared to other EVs and are 500–4000 nm in size [51]. Apoptotic bodies are distinct from exosomes and MVs because they contain cell organelles within them [15].

Since EVs are found in body fluids, especially blood and saliva, they are important sources of diagnostic and prognostic biomarkers [52,53]. EVs comprise a wide range of RNAs, such as messenger RNA (mRNA), ribosomal RNA (rRNA), long noncoding RNA (lncRNA), circular RNA, small nucleolar RNA, small nuclear RNA (snRNAs), transfer RNA, microRNA (miRNA), and piwi-interacting RNA (piRNA) [54,55]. Many studies have reported the use of EV-derived miRNAs in diagnostic, prognostic, and therapeutics fields [52,56,57]. Similar to proteins, miRNAs also show a cell origin signature, and miRNA levels in EVs vary from cell to cell. Mesenchymal stem cell (MSC)-derived EVs are enriched in proangiogenic miRNAs, such as miR-210 and miR-126 [20,43].

Some EVs contain DNAs, such as single-stranded DNA (ssDNA), mitochondrial DNA (mtDNA), and double-stranded DNA (dsDNA) [58]. They may range in size from 100 to several thousand base pairs [59] and, sometimes, even 2 million base pairs [60]. Little is known about the mechanisms of DNA packaging or the selective sorting of DNA into EVs (Figure 1). Better knowledge of EV biogenesis, biology, and contents might help us develop efficient and safe EV-based delivery nanoplatforms.

## 3. Engineering of EVMs

### 3.1. EVMs

**Figure 2:**
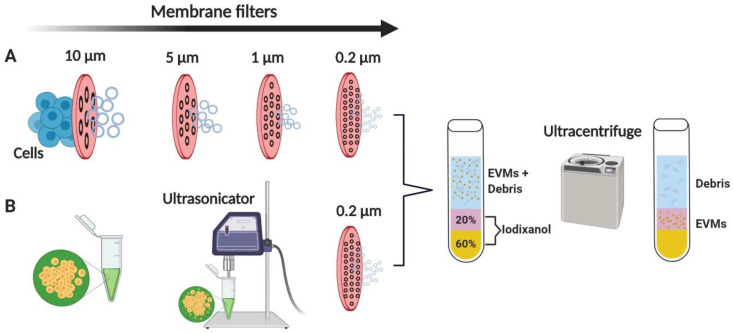
Schematic diagram of generating EVMs. (**A**) Cells in suspension are extruded through a polycarbonate membrane using a mini-extruder, and (**B**) cells in suspension are ultrasonicated for 1 min. Crude EVMs are filtered through a 0.2 µm filter to produce EMVs smaller than 200 nm. EMVs are purified by two-step OptiPrep density gradient ultracentrifugation. EVMs: extracellular vesicle mimetics. Figure created with BioRender.

Studies commonly use naturally secreted EVs as systems to deliver therapeutic agents in the treatment of several diseases. However, preclinical and clinical research is limited because EVs are produced by cells in low quantity [29,61]. To overcome this limitation, researchers have developed a method of generating EVMs from cells [28,29,31,62]. The large-scale production of EVMs might enable their use in a clinical setting. EVMs are created by the extrusion of live cells though a series of micrometer-sized membranes and filtration, and they are isolated from the interface between 20% and 50% iodixanol layers in two-step density gradient ultracentrifugation [29] (Figure 2A). An alternative way of producing EVMs from MSCs is via ultrasonication. Wang et al. (2019) used 1 min ultrasonication for delivery of shearing force to intact MSCs, followed by centrifugation to produce EVMs [63] (Figure 2B). With the same number of cells, ~20-to-100-fold more EVMs can be generated than naturally secreted EVs [29,62]. The advantages of EVM- over EV-based drug delivery systems with regard to clinical translation are listed in Table 1.

### 3.2. Hybrid EVMs

**Figure 3:**
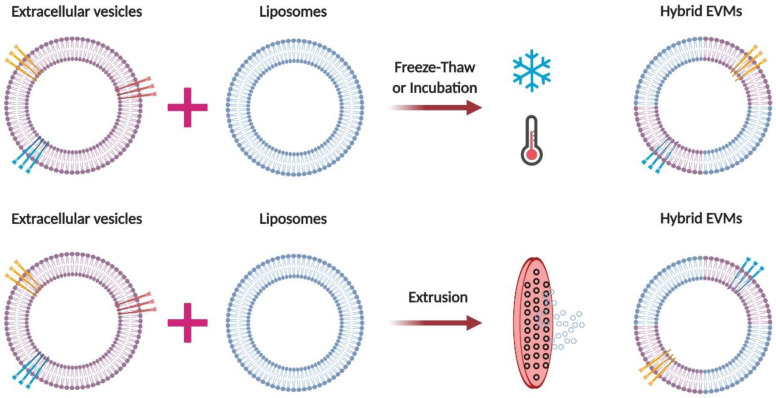
Schematic diagram of generating hybrid EVMs. (**Top**) Hybridization of EVs with synthetic liposomes with a freeze—thaw cycle. (**Bottom**) Hybridization of EVs with synthetic liposomes using membrane extrusion. EVMs, extracellular vesicle mimetics; EV, extracellular vesicle. Figure created with BioRender.

Hybrid EVMs comprise EV components and synthetic liposomes. The advantage of hybrid EVMs is that they consist of a lipid bilayer embedded with EV membrane proteins. This gives them specific properties of EVs that are favorable for therapeutic applications. In addition, liposomes can be easily modified (lipid modulation and flexible decoration with targeting) on the basis of treatment and loading approaches. Numerous methods are used to generate hybrid EVMs, such as freeze–thaw [65], incubation [66,67], and extrusion [68] (Figure 3). Hybrid EVMs could be a better alternative to EVs and liposomes as drug delivery systems by combining the advantages of both.

## 4. EV Internalization and Delivery of Materials into Cells

**Figure 4:**
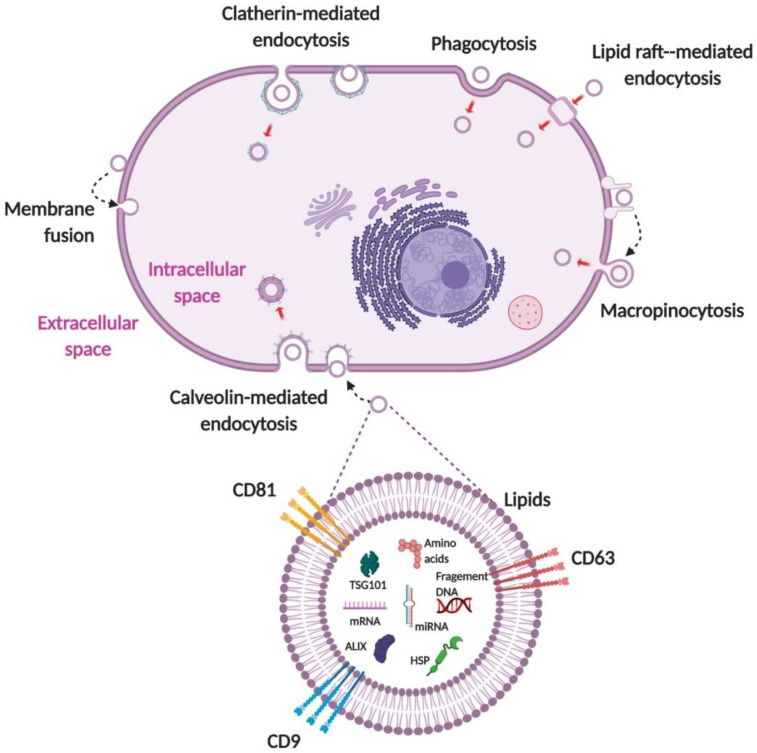
Internalization of EVs. EVs are internalized into cells through macropinocytosis, clatherin or calveolin-mediated endocytosis, lipid raft-mediated endocytosis, fusion, and phagocytosis. EVs deliver their cargo (proteins, RNAs, and DNAs), which is released into the cytoplasm or ER. EV, extracellular vesicle; ER, endoplasmic reticulum. Figure created with BioRender.

To deliver biological materials, loaded drugs, or nucleic acids into recipient cells, EVs must navigate through the plasma membrane. EVs are internalized into cells by various ways, such as macropinocytosis, clatherin or calveolin-mediated endocytosis, phagocytosis, and lipid raft-mediated and direct fusion (Figure 4) [69,70]. EV internalization is generally reported as an active process that uses a single or a combination of classical endocytic pathways [20,21,46,71]. Several studies have shown that EV cargo can be functionally delivered [12,22,30,46]. However, there is lack of proof and there are lack of rigorous protocols to explore cargo delivery [72]. It is unclear whether a different subpopulation of EVs internalized by recipient cells results in different localization, degradation, and/or functional outcomes of EV cargo [3]. The inconsistency in results might be because of the different types of cells used. Therefore, studies with specific mechanistic approaches are needed in order to completely elucidate EV internalization. We presume that like EVs, EVMs are internalized by various ways, but comparative studies are warranted.

## 5. Drug Loading into EVs and EVMs

### 5.1. Incubation

The incubation of drugs with EVs is a straightforward method of loading drugs into EVs. A drug is incubated at a specific temperature for a specific time, followed by purification and isolation. Sun et al. (2010) incubated curcumin with lymphoma cell line (EL-4)-derived exosomes for 5 min at room temperature (RT; 22 °C) and then performed sucrose gradient centrifugation. Successful curcumin loading was confirmed by high-performance liquid chromatography. The anti-inflammatory effects of curcumin-loaded exosomes were tested by using the murine macrophage cell line (RAW 264.7) in vitro and the lipopolysaccharide (LPS) mouse septic shock model in vivo; the results showed that exosomal curcumin has an increased anti-inflammatory activity compared to free curcumin [73].

Munagala et al. (2016) reported the loading of various drugs into exosomes isolated from bovine milk by incubation at RT, followed by the isolation of the drug-loaded exosomes using ultracentrifugation. Successful drug loading was confirmed spectrophotometrically and/or with ultraperformance liquid chromatography (UPLC). Drug-loaded exosomes showed significantly higher anticancer effects compared to the free drug against various human cancer cells in vitro and against lung tumor xenografts in vivo [74].

Agrawal et al. (2017) also used exosomes derived from bovine milk for drug loading. Exosomes were incubated with paclitaxel (PTX) for ~15 min at RT, and PTX-loaded exosomes were isolated by ultracentrifugation. Successful PTX loading was confirmed by UPLC. The practical loading efficiency was ~8%. Orally delivered and intraperitoneally-injected PTX-loaded exosomes showed 60% and 31% tumor growth, respectively, against human lung tumor xenografts in nude mice. Furthermore, PTX-loaded exosomes demonstrated remarkably lower systemic and immunologic toxicity compared to intravenously-injected free PTX [75].

Saari et al. (2015) first isolated EVs from prostate cancer cell lines LNCaP and PC-3 PCa and incubated them with PTX for 1 h at RT. PTX-loaded EVs were isolated by ultracentrifugation, and successful PTX loading was confirmed by UPLC. PTX cytotoxicity was enhanced by EV-mediated delivery into both LNCaP and PC-3 PCa cells. The results showed that the cancer cell-derived EVs could efficiently carry PTX to their parental cells and increase their cytotoxicity [76].

Qu et al. (2018) reported the isolation of exosomes from blood serum samples from the orbit venous the plexus of Kunming mice. The isolated exosomes were incubated with a dopamine solution with for 24 h at RT, and ultracentrifugation was performed to remove free dopamine. Successful dopamine loading was confirmed by liquid chromatography followed by tandem mass spectrometry (LC-MS/MS), and the quantity of dopamine in exosomes was measured. Dopamine-loaded exosomes showed a better therapeutic efficacy in a Parkinson’s disease (PD) mouse model and a lowered systemic toxicity compared to free dopamine. The results suggested that blood exosomes can be a promising drug delivery carrier [77].

Aqil et al. (2016) isolated bovine milk exosomes and incubated the exosomes with celastrol at RT. The free celastrol was removed, and celastrol-loaded exosomes were collected by ultracentrifugation. Celastrol loading was confirmed by UPLC. Celastrol-loaded exosomes showed a higher cytotoxicity to lung cancer (A549 and H1299) in a time- and concentration-dependent manner, as well as an increased antitumor efficacy against lung cancer cell xenografts with low or no systemic toxicity compared to free celastrol [78].

Kim et al. (2016) loaded PTX into exosomes with incubation, electroporation, and sonication, but PTX loading was significantly higher by sonication compared to incubation and electroporation: incubation at RT < electroporation < sonication. Therefore, sonication was selected for drug loading into exosomes [79].

Haney et al. (2015) isolated exosomes from macrophages and used five different methods (incubation, saponification, freeze–thaw, sonication, and extrusion) to load catalase into the exosomes. Catalase loading was lowest in incubation at RT compared to other methods. Exosomes loaded with catalase by incubation at RT also showed the lowest neuroprotective activity in vitro compared to exosomes loaded with catalase by other methods. In addition, the size of exosomes increased after catalase loading by all other methods except incubation, exosome aggregation occurred by the freeze–thaw method, and exosome deformation occurred during the sonication [80].

Goh et al. (2017) reported doxorubicin (Dox) loading in monocyte-derived EVs by incubation at 37 °C for 5 min, saponification for 5 min, incubation for 24 h at RT, and three freeze–thaw cycles. The highest Dox loading was observed by incubation at RT, followed by the saponification and freeze–thaw methods. Dox-loaded EVs showed higher cytotoxicity against both cancer and normal cells after incubation at 37 °C for 5 min compared to the free drug [81].

### 5.2. Sonication

The mechanical shear force produced by sonication compromises the membrane integrity of EVs, resulting in effective drug loading. Kim et al. (2016) loaded PTX into purified exosomes by sonication (six cycles of 30 s on/off for a total of 3 min, with 2 min cooling). They also loaded PTX with incubation and electroporation, but the loading efficiency was significantly higher with the sonication compared to incubation and electroporation, so for further experiments, the authors used sonication to load PTX into exosomes. PTX-loaded exosomes preferentially accumulated in lung cancer cells, with an efficient delivery of the drug into target cancer cells. In addition, PTX-loaded exosomes evaded the drug-resistant protein (P-glycoprotein-1)-mediated PTX efflux in resistant cancer cells more than free PTX did. These results showed that exosomes loaded with drugs are efficient in treating multidrug-resistant cancer cells [79].

Haney et al. (2015) isolated exosomes from macrophages and used five different methods (incubation, saponification, freeze–thaw, sonication, and extrusion) to load catalase into exosomes. Catalase loading increased with incubation at RT, followed by saponification < freeze–thaw < sonication, and a catalase activity assay confirmed catalase loading into exosomes. Exosomes loaded with catalase by sonication effectively accumulated in neurons and microglial cells in the brain, and they produced a potent neuroprotective effect compared to free catalase in a PD mouse model [80].

Lamichhane et al. (2016) isolated EVs from kidney cells and successfully loaded small RNAs into EVs by sonication. EVs loaded with siRNA by sonication were readily taken up by incubated cells and induced mRNA knockdown, eventually leading to low target protein expression [82].

### 5.3. Electroporation

In electroporation, EVs are suspended in a conductive solution with drugs and exposed to an electrical field that temporarily disrupts their phospholipid membranes, leading to temporary pore formation. Drugs or nucleotides can subsequently enter the EVs. This method is widely used for loading nucleotides.

Usman et al. (2018) used human red blood cell (RBC)-derived EVs from group O blood samples. The electroporation of the EVs with anti-miR-125b antisense oligonucleotides (ASOs), Cas9 mRNA, anti-miR-125b, and gRNA (genomic RNA) was performed, and the successful loading of nucleic acids was confirmed by polymerase chain reaction or Western blotting. RBC-derived EVs have been found to deliver RNA drugs, including ASOs and Cas9 mRNA, and guide RNAs into both human cells and xenograft mouse models. RNA drug delivery with RBC-derived EVs has shown highly robust microRNA inhibition and CRISPR-Cas9 genome editing in both human cells and xenograft mouse models, with no observable cytotoxicity [83].

Kim et al. (2016) showed the successful loading of PTX into purified exosomes by incubation, electroporation, and sonication. PTX loading increased as follows: incubation at RT < electroporation < sonication. Thus, for further experiments, the authors used PTX-loaded into exosomes by sonication [79].

Tian et al. (2014) loaded Dox into DC-derived exosomes with integrin-specific Arg-Gly-Asp (iRGD) by electroporation. Dox-loaded exosomes were isolated by ultracentrifugation, and Dox loading was quantified by fluorescence spectrophotometry. Dox-loaded iRGD exosomes showed a higher cytotoxicity to various human cancer cells compared to Dox-loaded blank exosomes. Mice bearing MDA-MB-231 tumors were administered an intravenous injection of Dox-loaded iRGD exosomes, leading to tumor growth inhibition without overt toxicity [84].

### 5.4. Freeze–Thaw

The freeze–thaw method is a straightforward technique used to load drugs into EVs. EVs are mixed with drugs, and then they undergo a few cycles of freezing at −80 °C in liquid nitrogen and thawing at RT. Haney et al. (2015) loaded catalase into exosomes, as mentioned before. Catalase loading increased more by the freeze–thaw method than straightforward incubation at RT, although the loading level was moderate. However, repeating freeze–thaw cycles can lead to the degradation of many EV proteins and structural changes in EVs [80].

### 5.5. Extrusion

Haney et al. (2015) loaded catalase into exosomes, as mentioned before. Catalase loading increased by incubation at RT, followed by freeze–thaw < sonication = extrusion, as observed by Western blotting. Extrusion showed high levels of loading, while sonication and extrusion showed greater neuroprotective activity in vitro compared to the freeze-thaw method or incubation at RT. However, the size of exosomes increased, and exosomes showed deformation by extraction compared to incubation [80].

Kalimuthu et al. (2018) reported a new approach of loading PTX into EVMs. Human bone marrow-derived MSCs were mixed with PTX and subjected to serial extrusion and filtration. Loaded EVMs were isolated by two-step OptiPrep density gradient ultracentrifugation. PTX-loaded EVMs significantly decreased the viability of breast cancer cells in vitro and significantly inhibited in vivo tumor growth compared to controls and/or EVMs [30].

Lu et al. (2018) engineered hybrid EVMs by mixing various lipid compositions similar to exosomes, and they prepared a lipid film. The lipid film was mixed with vascular endothelial growth factor (VEGF) siRNA and extruded to generate hybrid EVMs; VEGF siRNA was similarly loaded into liposomes. The siRNA-loaded hybrid EVMs and liposomes were isolated by ultrafiltration. The siRNA-loaded hybrid EVMs exhibited significantly higher cellular uptake and efficient gene silencing compared to siRNA-loaded liposomes [85].

Lunavat et al. (2016) generated EVMs through the serial extrusion of cells transduced with short hairpin RNA (shRNA) through nanosized filters, and they isolated them by two-step OptiPrep density gradient ultracentrifugation. The siRNA-loaded EVMs were taken up by recipient cells, and siRNA induced functional knockdown responses in them, resulting in the attenuation of the target gene (*c-Myc*), eventually leading to the activation of apoptotic markers, such as cleaved poly(ADP-ribose) polymerase (PARP) and cleaved caspase 3 [86].

Tao et al. (2018) generated EVMs through the serial extrusion of cells transduced with the lncRNA-H19 Smart Silencer (H19-SS) through nanosized filters, and they isolated them by two-step OptiPrep density gradient ultracentrifugation. H19-SS-EVM-loaded sodium alginate hydrogel treatment increased endothelial cell proliferation and tube formation compared to negative control EVMs. H19-SS-EVMs could defuse hyperglycemia and accelerate healing in a diabetic rat chronic wound model [87].

### 5.6. Saponification

Saponin is used as a membrane permeabilizer to assist cargo loading. It is a surface-active agent and induces the formation of small pores within lipid membranes, which allows drugs or other molecules to enter EVs. Haney et al. (2015) loaded catalase into exosomes, as mentioned before. Saponification-based catalase-loaded exosomes were used to assess the therapeutic effect on a PD mouse model. Exosomes loaded with catalase by saponification effectively accumulated in neurons and microglial cells in the brain, and they produced a potent neuroprotective effect compared to sonication [80].

Fuhrmann et al. (2015) used endothelial, cancer, and stem cells to isolate EVs. Porphyrin was loaded into the EVs by saponification, electroporation, extrusion, and dialysis. Porphyrin loading was good, regardless of the loading method used. More porphyrins were loaded into EVs compared to liposomes. Saponin treatment enhanced porphyrin loading into EVs and also cellular uptake compared to the other four methods [88].

### 5.7. Transfection Reagents

The commercially available transfection reagents are generally used for the transfection of nucleic acids to cells [89,90]. Wahlgren et al. (2012) demonstrated loading of siRNA to human plasma derived exosomes by transfection reagents. They incubated the exosomes with siRNA (MAPK-1) along with a transfection reagent for 10 min at RT, and they isolated the exosomes by aldehyde/sulfate latex beads to remove the excess of micelles. The successful loading and delivery of functional siRNA was confirmed by flowcytometry and/or Northern blotting [91].

Shtam et al. (2013) reported the loading of siRNA into exosomes derived from fibrosarcoma cells. They also incubated the siRNA (RAD51 or RAD52) with a transfection reagent for 10 min at RT, and then this mixer was incubated with exosomes for 30 min at RT. Exosomes were filtered through a 100-kDa filter to remove the excess of micelles. The loading of siRNA was confirmed by flowcytometry and fluorescent microscopy. The siRNA loaded with exosomes induced the apoptosis of cancer cells [92].

### 5.8. Drug Treatment of Parental Cells

The drug treatment of parental cells is an alternative approach to loading drugs into EVs before they are secreted into condition media (CM) and isolated. Jang et al. (2013) incubated cells with Dox and then extruded them using serial extrusion through filters with diminishing pore sizes. Dox-loaded EVMs were isolated by two-step OptiPrep density gradient ultracentrifugation. Dox-loaded EVMs moved into the tumor and inhibited tumor growth without side effects, as compared to the equipotent free drug [62].

Pascucci et al. (2014) incubated PTX with murine MSCs for 24 h and then isolated EVs from the CM of PTX-incubated and non-PTX-incubated MSCs. The antiproliferative effects of CM and MSC-PTX were tested on human pancreatic adenocarcinoma. MSC-PTX-derived EVs showed strong antiproliferative activity against human pancreatic adenocarcinoma. This study first demonstrated how active drugs can be packed and delivered through EVs in vitro through the direct incubation of drugs with MSCs [93].

### 5.9. Gene Engineering of Parental Cells

To load small RNAs and proteins into EVs, cells are transfected with miRNA or target/therapeutic protein-coded plasmids and EVs are isolated from those cells. This is believed to be a sophisticated drug-loading methodology. Li et al. (2019) transduced cells with CD9 and an RNA-binding protein (CD9-R; known to bind with miR-155), and then they isolated exosomes from those cells. CD9-R successfully enriched miR-155 into exosomes. The miR-155-enriched exosomes were efficiently delivered into recipient cells, and they recognized endogenous targets (Socs1) and inhibited protein expression. The intravenous administration of miR-155-enriched exosomes into mice decreased Socs1 expression in the liver, spleen, lungs, and kidneys [94].

Sterzenbach et al. (2017) reported that Nedd4 family interacting protein 1 (Ndfip1) interacts with the WW domains of Nedd4 family ubiquitin ligases through three L-domain motifs. They transduced cells with WW-Cre and found that exosomes with WW-Cre expression induce DNA recombination, demonstrating the functional delivery of the protein to recipient cells. In addition, the authors investigated the nasal route for exosomal protein delivery to brain tissue in vivo. The results demonstrated that exosomes deliver protein to brain regions by trespassing the blood–brain barrier [95].

**Figure 5:**
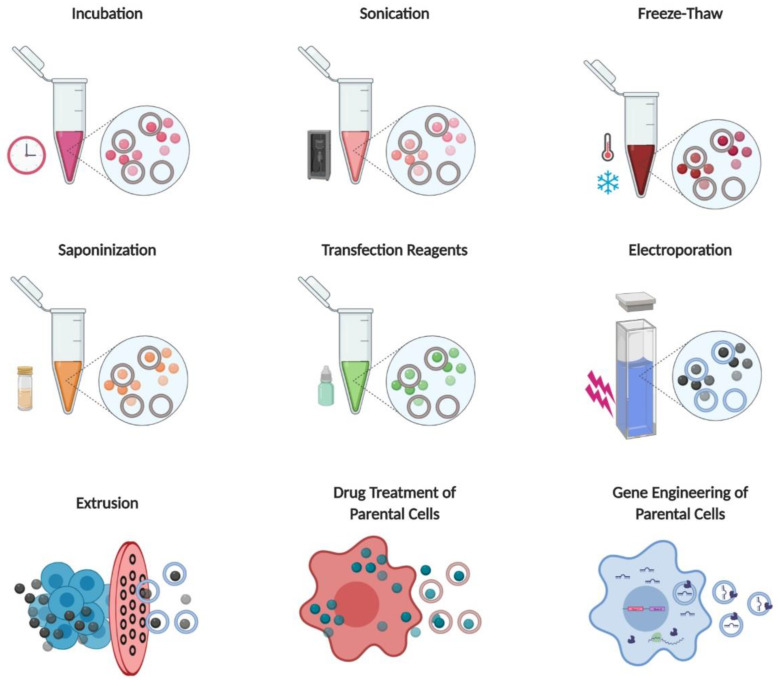
Illustration of methods of loading therapeutics into EVs and EVMs. Starting from the top, left to right: incubation, sonication, freeze-thaw, saponification, transfection reagents, electroporation, extrusion, parent cell treatment with drugs, and the gene engineering of parental cells. EV, extracellular vesicle; EVMs, extracellular vesicle mimetics. Figure created with BioRender.

Cho et al. (2018) transduced cells with signal regulatory protein α (SIRPα, a CD47 antagonist) plasmid and isolated exosomes with SIRPα expression. They injected these exosomes into tumor-bearing mice by intratumoral injection. Exosomes with SIRPα expression showed higher tumor growth inhibition compared to the same dose of protein-scaffold-based nanocages, such as ferritin-SIRPα-nanocages [96]. Table 2 and Figure 5 present all different loading methods with examples of EVs as drug delivery systems.

## 6. Advantages and Disadvantages of EV- and EVM-Based Drug Loading

EVs may have advantages over synthetic drug delivery systems because of their intrinsic targeting capabilities, tetraspanin surface proteins, and immune-escape properties [12]. The advantage of using incubation for drug loading is that it is the simplest method and does not require extra equipment or any kind of solution for drug loading. Compared to other methods, incubation does not affect the size and morphology of EVs. However, one disadvantage is its lack of drug-loading capacity [73,79,80,81,88].

Sonication shows an increased drug-loading capacity compared to other methods. It can be used to load various drugs, such as anticancer drugs, siRNAs, and proteins, but it is unsuitable for hydrophobic drugs [79,80,82]. Electroporation can be used to load large molecules, such as nucleic acids (e.g., ASOs, siRNA, mRNA, and gRNA), and anticancer drugs. Electroporation shows a relatively higher drug-loading capacity compared to incubation. However, it has a few disadvantages such as EV deformation, a low drug-loading capacity compared to sonication or saponification, and siRNA aggregation [64,79,82,83,88,97].

The freeze-thaw method is also a straightforward method used to load drugs into EVs and EVMs. It has a relatively moderate drug-loading capacity. During freezing and thawing, membrane fusion is possible, so the freeze-thaw method is used in making hybrid EVMs from EVs and liposomes. This method also has a few disadvantages such as a low drug-loading efficiency compared to extrusion and sonication, in addition to EV aggregation [80,81].

Saponin is used as an “assistant” during loading drugs into EVs. Saponification permeabilize EVs to load drugs by mixing the saponin reagent with drugs to EVs. It has a higher drug-loading capacity compared to mixing or incubation. However, saponin generates pores on EV membranes, so the process requires a recovery phase. In addition, saponin is a toxic agent [98], so EVs require additional washing, which might affect their integrity. Using saponin treated-EVs as a control is a must in vitro or in vivo treatment to eliminate the toxicity of saponin from treatment effects, and saponin also gets loaded along with drugs into EVs [80,81,88].

The loading of RNAs into EVs with transfection reagents, a straightforward method of mixing RNAs, does not require additional equipment. The disadvantages of using transfection reagents are their expensive cost of agents and their not well-known loading mechanisms. In addition, free RNAs or complexes may be co-isolated with EVs (high centrifugal forces), so stringent procedures are required for remove their contamination. Moreover, transfection reagent residues complexed with RNAs may affect the function of siRNAs [91,92,99].

Extrusion is used for the large-scale production of EVMs required for clinical application and is also used to produce hybrid EVMs from EVs and liposomes (Table 1). Extrusion has the highest drug-loading capacity for any kind of drug compared to other methods. However, it has the disadvantage of membrane deformation [80,81,85,86,87,97].

Another simple but less reported method of loading drugs into EVs is treating cells with drugs and isolating EVs. However, this method has a low drug-loading capacity and could harm cells, leading to the release of unwanted materials into EVs and the contamination of isolated EVs by apoptotic bodies [93].

The engineering the gene of parental cells and isolating EVs guarantee that therapeutic molecules are already loaded into EVs, even before secretion, so drug-loading efficacy is high [94]. This method also shows a high packaging efficiency, although the underlying mechanism is unclear [94,95].

Every method discussed in this report has advantages and disadvantages (Table 3), and any method can be selected depending on the type of drug delivery application.

## 7. Clinical Development and Future Prospects

The drug loading and delivering properties of EVs and the substantially successful preclinical results have encouraged researchers to experiment within the development of EV-based therapies and EV-based drug delivery platforms. Initially, the two phase-I clinical trials were tested to see the feasibility and safety of exosomes derived from DCs pulsed with antigenic peptides on metastatic melanoma and non-small lung cancer patients. The therapeutic outcome of these two studies were promising for further studies [27,100]. A phase-II study with Interferon -γ-DCs pulsed with peptides was tested on non-small lung cancer patients; the results of the study were promising as it enhanced the natural killer cells’ antitumor activity, and 32% of patients achieved progression-free survival (four months) [101]. These studies have shown that EVs can be used in humans without life-threatening complications and have paved the way for EV-based therapies in clinics. The feasibility and safety of EVs as a drug delivery system should being tested in a clinical setting because there only a few clinical trials have been completed [32]. Recently, four clinical studies were proposed to deliver drugs using EV to certain diseases. These studies will load drugs (curcumin, chemotherapeutic drugs, miR124, and KRAS siRNA) and deliver them to diseases (colon cancer, malignant pleural effusion, acute ischemic stroke, and metastatic pancreatic cancer) [102,103,104,105]. These four studies are recruiting or going to recruit patients, and the outcomes of these studies will open doors for the EV-based drug delivery field.

Extensive research on the various methods of making EVMs (or hybrid EVMs) as potential drug carriers is being carried out, and these methods need to be standardized, a process that is extremely challenging in this emerging EV-based nanocarrier field. Nevertheless, we believe that in the near future, advancements in our knowledge of the biogenesis and biological properties of EVs, together with rapid advances in hybrid EVM technology, will lead to a novel class of safe and efficient EVs and EVMs for drug delivery.

## Figures and Tables

**Table 1:** Advantages and disadvantages EVs and EVMs.

	Advantages	Disadvantages	References
**Natural EVs**	Natural packing of biological materials (e.g., protein and RNAs) Defined biomarkers (CD63, CD9, CD81, and Alix)	Low yield Isolation—time consuming Low/moderate loading capacity	[1,2,29,30,32,62,64]
**Engineered EVMs**	High yield (generally 100-fold more than EVs) Isolation—short time (20-fold-less than EVs) High loading capacity Generation of Hybrid EVMs	Deformation of membrane Contamination of DNAs Random packing of biological materials No definite biomarker	[25,28,29,32,65,66]

**Table 2:** Different loading methods with examples of EVs and EVMs as drug delivery systems.

Drug-Loading Method	Drug/Agent	Type of EVs	EVs Origin	In Vitro or In Vivo	Disease Target	Outcome	Reference
**Incubation**	Curcumin	Exosomes	Lymphoma cells	In vivo (mice)	Inflammation	Anti-inflammatory	[73]
	Paclitaxel	Exosomes	Bovine milk	In vivo (mice)	Cancer	Inhibition of cancer	[74]
	Docetaxel	Exosomes	Bovine milk	In vivo (mice)	Cancer	Inhibition of cancer	[74]
	Withaferin A	Exosomes	Bovine milk	In vivo (mice)	Cancer	Inhibition of cancer	[74]
	Paclitaxel	Exosomes	Bovine milk	In vivo (mice)	Cancer	Inhibition of cancer	[75]
	Paclitaxel	EVs	prostate cancer cell	In vitro	Cancer	Inhibition of cancer	[76]
	Dopamine	Exosomes	Blood	In vivo (mice)	Parkinson’s disease	Better therapeutic efficacy and lower systemic toxicity	[77]
	Celastrol	Exosomes	Bovine milk	In vivo (mice)	Cancer	Inhibition of cancer and lower systemic toxicity	[78]
	Catalase	Exosomes	Macrophage cells	In vitro	Parkinson’s disease	Neuroprotective effects	[80]
	Paclitaxel	Exosomes	Macrophage cells	In vivo (mice)	Cancer	Inhibition of cancer	[79]
	Doxorubicin *	EVMs	monocytes	In vitro	Cancer	Inhibition of cancer	[81]
	Doxorubicin **	EVMs	monocytes	In vitro	Cancer	Loading was successful	[81]
**Sonication**	Paclitaxel	Exosomes	Macrophage cells	In vivo (mice)	Cancer	Inhibition of cancer	[79]
	Catalase	Exosomes	Macrophage cells	In vivo (mice)	Parkinson’s disease	Neuroprotective effects	[80]
	siRNA/miRNA/ssDNA	EVs	Kidney cells	In vitro	Cells	knockdown of gene expression	[82]
**Electroporation**	126b-ASO/Cas9 mRNA/gRNA	EVs	RBCs	In vitro or In vivo (mice)	Cancer	Inhibition of cancer	[83]
	Paclitaxel	Exosomes	Macrophage cells	In vivo (mice)	Cancer	Inhibition of cancer	[79]
	BACE-1 siRNA	Exosomes	BMDCs	In vitro or In vivo (mice)	Mouse brain	knockdown of gene expression	[97]
	Doxorubicin	Exosomes	DCs	In vitro or In vivo (mice)	Cancer	Inhibition of cancer	[84]
	Porphyrins	EVs	endothelial, cancer and stem cells	In vitro	Cells	Cellular uptake was higher than liposomes	[88]
	siRNA/miRNA/ssDNA	EVs	Kidney cells	In vitro	Cells	knockdown of gene expression	[82]
**Freeze-Thaw**	Catalase	Exosomes	Macrophage cells	In vitro	Parkinson’s disease	Neuroprotective effects	[80]
	Doxorubicin	EVMs	monocytes	In vitro	Cancer	Loading was successful	[81]
**Saponification**	Catalase	Exosomes	Macrophage cells	In vivo (mice)	Parkinson’s disease	Neuroprotective effects	[80]
	Porphyrins	EVs	endothelial, cancer and stem cells	In vitro	Cells	Cellular uptake was higher than liposomes	[88]
	Doxorubicin	EVMs	monocytes	In vitro	Cancer	Loading was successful	[81]
**Transfection reagents**	MAPK-1 siRNA	Exosomes	Plasma	Invitro	Normal Cells	knockdown of gene expression	[91]
	RAD51 or RAD52 siRNA	Exosomes	Cancer cells	Invitro	Cancer	knockdown of gene expression	[92]
**Extrusion**	Catalase	Exosomes	Macrophage cells	In vitro	Parkinson’s disease	Neuroprotective effects	[80]
	Paclitaxel	EVMs	MSCs	In vitro or In vivo (mice)	Cancer	Inhibition of cancer	[30]
	VEGF siRNA	Hybrid EVMs	Lipid composition of exosomes	In vitro	Cancer	Inhibition of cancer	[85]
	c-Myc SiRNA	Nanovesicles	Fibroblast	In vitro	Cancer	Inhibition of c-Myc protein and activation of apoptosis	[86]
	LncRNA-H19 Smart Silencer (H19-SS)	EVMs	Kidney cells	In vitro or In vivo (rat)	Diabetic wound model	Accelerate the healing processes	[87]
	Porphyrins	EVs	endothelial, cancer and stem cells	In vitro	Cells	Cellular uptake was higher than liposomes	[88]
**Drug Treatment of Parental Cells**	Doxorubicin	EVMs	Macrophage	In vitro or In vivo (mice)	Cancer	Inhibition of cancer and lower systemic toxicity	[62]
	Paclitaxel	MVs	MSCs	In vitro	Cancer	Anti-Proliferation	[93]
**Gene Engineering of Parental Cells**	miR-155	Exosomes	Kidney cells and murine liver cells	In vitro or In vivo (mice)	Normal Cells and naïve mice	knockdown of gene expression	[94]
	Ndfip1	Exosomes	Kidney cells	In vitro or In vivo (mice)	Cancer cells and naïve mice	Inducing DNA recombination	[95]
	SIRPα	Exosomes	Kidney cells	In vitro or In vivo (mice)	Cancer	Increased targeting and inhibition of cancer	[96]

* Incubation for 5 min at 37 °C; ** Incubation for 24 h at RT (22 °C); EV, extracellular vesicle; EVMs, extracellular vesicle mimetics; siRNA, small interfering RNA; miRNA, microRNA; ssDNA, single-stranded DNA; gRNA, genomic RNA; lncRNA, long noncoding RNA; ASO, antisense oligonucleotide; VEGF, vascular endothelial growth factor; Ndfip1, Nedd4 family interacting protein 1; SIRPα, signal regulatory protein α; RT, room temperature.

**Table 3:** Advantages and disadvantages of different drug-loading methods.

Drug-Loading Method	Advantage	Disadvantage	Reference
**Incubation**	Simple No additional equipment required Not affect the EVs (size and morphology)	Low loading capacity	[73,79,80,81,88]
**Sonication**	High loading capacity, Able to load anticancer drugs, siRNAs and proteins	Not suitable for hydrophobic drugs	[79,80,82]
**Electroporation**	Able to load large molecules (nucleic acids such as antisense oligo nuclides, siRNA, miRNA and genomic RNA) and anticancer drugs Moderate loading capacity	Deformation the EVs siRNA aggregation Low loading capacity compared to sonication or saponin	[64,79,82,83,88,97]
**Freeze-Thaw**	Simple Moderate of loading capacity Membrane fusion is possible: Generation of Hybrid EVMs from EVs and liposomes	Low loading efficiency compared to extrusion and sonication Aggregation of EVs	[80,81]
**Saponification**	Simple Higher drug-loading capacity	Requires a recovery phase before use Saponin is a toxic agent Requires additional washing (affect the integrity of EVs) Using a saponin control-EVs is must	[80,81,87,98]
**Transfection reagents**	Simple Able to load nucleic acids	Expensive siRNA also isolated with EVs (high centrifugal forces) Transfection reagent may associate with siRNA and delivered into recipient cells Mechanism of action for reagents not well-known	[91,92,99]
**Extrusion**	Highest loading of any kinds of drugs Generation of Hybrid EVMs from EVs and liposomes	Deformation of membrane	[80,81,85,86,87,97]
**Drug Treatment of Parental Cells**	Simple No Additional Equipment Required	Low loading capacity Loading may harm the cells Cells lead to release unwanted materials into EVs. Activation of apoptotic bodies may contaminate the isolated EVs	[93]
**Gene Engineering of Parental Cells**	High packaging efficiency Guaranteed loading	How it works remain elusive Mostly used to load nucleic acids	[94,95]

EV, extracellular vesicle; EVMs, extracellular vesicle mimetics; siRNA, small interfering RNA; miRNA, microRNA.

## Abbreviations

Dox	Doxorubicin
dsDNA	Double-stranded DNA
EVMs	Extracellular vesicle mimetics
EVs	Extracellular vesicles
gRNA	Genomic RNA
ILVs	Intraluminal vesicles
lncRNA	Long noncoding RNA
miRNA	MicroRNA
MVB	Multivesicular body
Nedd4f1	Nedd4 family interacting protein 1
PTX	Paclitaxel
siRNA	small interfering RNA
Sirpα	Signal regulatory protein α
ssDNA	Single-stranded DNA
